# Sex- and Time-Dependent Patterns in Risk Factors of End-Stage Renal Disease: A Large Austrian Cohort with up to 20 Years of Follow-Up

**DOI:** 10.1371/journal.pone.0135052

**Published:** 2015-08-31

**Authors:** Constanze Pscheidt, Gabriele Nagel, Emanuel Zitt, Reinhard Kramar, Hans Concin, Karl Lhotta

**Affiliations:** 1 Agency for Preventive and Social Medicine, Bregenz, Austria; 2 Institute of Epidemiology and Medical Biometry, Ulm University, Ulm, Germany; 3 Department of Nephrology and Dialysis, Academic Teaching Hospital Feldkirch, Feldkirch, Austria; 4 Vorarlberg Institute for Vascular Investigation and Treatment, Academic Teaching Hospital Feldkirch, Feldkirch, Austria; 5 Austrian Dialysis and Transplant Registry, Rohr im Kremstal, Austria; University Medical Center Groningen and University of Groningen, NETHERLANDS

## Abstract

**Objective:**

We investigated the association between metabolic factors and End-Stage Renal Disease (ESRD) and quantified the magnitude of their influence dependent on sex and time of exposure up to 20 years.

**Material and Methods:**

A prospective cohort study was conducted to determine risk factors for the development of ESRD. From 1988 to 2005 185,341 persons (53.9% women) participated in the “Vorarlberg Health Monitoring and Promotion Programme” (VHM&PP). Data on body mass index (BMI), fasting blood glucose (FBG), systolic (BPsys) and diastolic (BPdia) blood pressure, total cholesterol (TC), triglycerides (TG), gamma-glutamyltransferase (GGT) and smoking status were collected. Data of the population-based VHM&PP were merged with the Austrian Dialysis and Transplant Registry. Cox proportional hazards models were applied to calculate hazard ratios (HRs) for ESRD, stratified by sex and 5-year time intervals.

**Results:**

During a mean follow-up of 17.5 years 403 patients (39.1% women) developed ESRD. Significant risk factors were: BMI (per 1 kg/m^2^) HR 1.04 (95% CI 1.01–1.06), FBG (per 1 mmol/L) HR 1.09 (1.05–1.12), BPsys (per 5 mmHg) HR 1.10 (1.07–1.14), BPdia (per 5 mmHg) HR 1.09 (1.03–1.15), TG (per 1 mmol/L) HR 1.07 (1.02–1.13), TC (per 1 mmol/L) HR 1.22 (1.13–1.32). We observed a sex-specific risk pattern with an increased ESRD risk for men for increasing TG and smoking, and for women for increasing BMI and GGT. In time interval analyses BPsys and TC were associated with early ESRD onset, whereas BMI, FBG, BPdia and GGT were associated with later onset.

**Conclusions:**

Anthropometric and metabolic factors are differentially associated with the long-term risk for ESRD in a sex- and time-dependent manner. Consideration of these patterns in preventive and therapeutic strategies could have an impact on ESRD incidence.

## Introduction

On a global scale the number of patients suffering from chronic kidney disease (CKD) and end-stage renal disease (ESRD) is increasing [1]. About one in 40 of middle aged men and one in 60 of women will develop ESRD during their lifetimes [2]. The risk for ESRD has been calculated as 1.1 in 10.000 patient-years for individuals without albuminuria [3]. Kidney diseases are associated with a large burden of morbidity and mortality, and the treatment of ESRD patients is expensive [4, 5]. CKD is often asymptomatic and may progress to ESRD when unrecognized. It is therefore highly desirable to identify risk factors that allow early detection of individuals who are at increased risk for developing CKD and ESRD later in life. Several factors such as age, male sex, diabetes, hypertension and obesity have been identified in previous studies [6–8]. However, not all of these studies were stratified by sex or considered in time interval analysis [6, 7] and they often included only a limited number of participants [8]. More recent studies have found metabolic factors to predict CKD up to thirty years before ESRD [9, 10]. These studies, however, were not performed in a population-based prospective cohort and did not describe a large study population. Other studies focused on non-Caucasian populations and were limited by a short follow-up [11–13]. A prior meta-analysis reported that the metabolic syndrome and its components are associated with the development of CKD [14]. Identification of such risk factors is valuable, because it would enable early preventive and therapeutic interventions in order to reduce the future development of CKD and progression to ESRD.

The Austrian Dialysis and Transplant Registry (OEDTR) was established by the Austrian Society of Nephrology and collects data on all patients who received chronic renal replacement therapy (RRT) in Austria since 1964 and has almost complete follow-up [15]. Since 1985 data on cardiometabolic risk factors are electronically available from adults living in Vorarlberg during health examinations (HE) as part of the Vorarlberg Health Monitoring and Prevention Programme (VHM&PP) [16] aiming at the prevention of chronic diseases and promoting public health [17]. Unfortunately, this data set does not include information on renal function or proteinuria. The OEDTR and the VHM&PP are both well characterised data sets and their linkage provides the unique opportunity to investigate the association of metabolic risk factors and to quantify the magnitude of sex- and time-dependent patterns in as large cohort with long-term follow-up.

## Methods

### Study Design

A prospective cohort study was conducted to determine risk factors for the development of ESRD. For this purpose from the Vorarlberg Health Monitoring and Prevention Program (VHM&PP) the Austrian Dialysis and Transplant Registry (OEDTR) were merged.

### Study Population

Between 01.01.1988 and 30.06.2005 185,367 persons participated in the VHM&PP [16] a population-based risk factor surveillance program in Vorarlberg, the westernmost province of Austria. Every adult in Vorarlberg above the age of twenty was invited via public media to undergo a health examination (HE) at their general practitioner. Data were collected prospectively including measurements of height and weight (with the participants wearing light indoor clothes and no shoes), systolic and diastolic blood pressure. Fasting blood samples were taken to determine blood glucose, triglycerides, total cholesterol and gamma-GT. Participants' smoking status was inquired and categorized in ‘never smokers’ and ‘ever smokers’.

### Definition of ESRD

The OEDTR collects data on all patients who have received chronic renal replacement therapy in Austria since 1964 and has almost complete follow-up [15]. ESRD is defined as initiation of renal replacement therapy, either dialysis or renal transplantation. The data are provided by the Austrian dialysis and transplant centres.

The analysed VHM&PP data set included 185,341 participants (99,881 women, 85,460 men). After merging the VHM&PP data and the OEDTR data, 429 patients with ESRD requiring dialysis or transplantation were detected. We excluded nine prevalent dialysis patients, and a further 17 patients due to an ESRD diagnose after the census date, namely 31.12.2009.

### Ethic statement:

Ethics approval was obtained from the Ethics Committee of the State of Vorarlberg.

All patients registered in the OEDTR signed a declaration of consent to permit their data to be transferred to the registry.

### Statistical Analysis

Outcome was defined as end-stage renal disease (ESRD) requiring dialysis or transplantation. Only data of the first health examination was included in the analysis. Follow-up began after the baseline health examination and ended at ESRD diagnosis, death or the censoring date (31.12. 2009), whichever occurred first.

To provide information on clinically relevant cut-points, we also calculated the models including categorical variables: Age (years) categories were defined as < 30; 30–39; 40–49; 50–59; 60–69; ≥ 70. BMI was calculated as weight in kilograms divided by squared height in meters. Categories for BMI (kg/m^2^) were defined according to the WHO [18]: ‘underweight’ (BMI < 18.5), ‘normal weight’ (BMI ≥ 18.5 and < 25), ‘overweight’ (BMI ≥ 25 and < 30) and ‘obese’ (BMI ≥ 30) [18]. Blood glucose levels (mmol/L) were termed ‘normal’ (<5.6), ‘prediabetes’ (≥ 5.6 and ≤ 6.9) and ‘diabetes’ (> 6.9) [19]. Blood pressure (BP in mmHg) was categorised as normal (BP systolic < 120 and BP diastolic <80), prehypertension (BP systolic 120–139 and BP diastolic 80–89), hypertension stage I (BP systolic 140–159 and BP diastolic 90–99), and stage II (BP systolic ≥ 160 and BP diastolic ≥ 100) [20]. Categories for triglycerides (mmol/L9 were designated ‘normal’ (< 1.2), ‘borderline high’ (≥ 1.2 and < 2.3), ‘high’ (≥ 2.3 and < 5.7) and ‘very high’ (≥ 5.7). Cholesterol (mmol/L) was categorised: ‘desirable’ (< 5.2), ‘borderline high’ (≥ 5.2 and < 6.2) and ‘high’ (≥ 6.2) [21]. For gamma-GT sex-specific cut-offs were used. For men elevated gamma-GT values were defined as ≥ 61 U/L, for women elevated gamma-GT values were defined as ≥ 36 U/L.

Cox proportional hazards models were fitted to calculate Hazard ratios (HRs) and 95% confidence intervals (CI) for ESRD. Wald test was used to test for interaction between pair wise exposure. Additional to continuous models, categorical data were used according to clinically meaningful cut-off points. The proportional hazard assumption for categoral exposure variables has been tested by Kaplan Meier curves. Due to skewness gamma-GT values were logarithmized. All models were adjusted for age, sex and smoking status (basic adjustment model). The fully adjusted model included all metabolic risk factors simultaneously and was adjusted for age, sex and smoking status. We tested the Cox proportionality assumption over time by creating interactions terms of the predictors and a function of survival time including them in the model. Overall, the proportionality test was < 0.001. HRs were calculated by censoring the follow-up time in 5-year time intervals (5 years 5–10 years, 10–15 years and more than 15 years after baseline) in order explore time-dependent associations. Interactions were tested and not found to influence the results. All calculations were performed using the statistical analysing software SAS, release 9.3 (SAS Institute, Cary, NC, USA).

## Results

A total of 185,341 participants (99,881 women and 85,460 men) were analysed in this study. Median age at baseline was 38.9 (Q1, Q3; 28.8; 52.4)) years and median follow-up time was 19.2 (Q1, Q3; 12.6, 22.8)) years. Of all participants, 31.1% were overweight, 10.8% were obese and 28.8% were smokers. A detailed description of all anthropometric and metabolic factors determined at baseline stratified by sex in participants with and without later development of ESRD is provided in Table 1.

**Table 1:** Baseline description of the study population.

		Women*								Men*							
	Total	No ESRD				ESRD				No ESRD				ESRD			
	n	n	Q1	Median	Q3	n	Q1	Median	Q3	n	Q1	Median	Q3	n	Q1	Median	Q3
**Age [years]**	185,341	99,723	28.1	38.6	52.8	158	43.4	53.6	61.7	85,215	29.5	39.1	51.9	245	43.7	51.5	61.3
**Follow-up [years]**	185,341	99,723	13.2	19.7	23.1	158	18.2	22.3	23.8	85,215	12.0	18.7	22.5	245	17.6	21.6	23.7
**Age at ESRD [years]**	403	-	-	-	-	158	56.1	64.6	73.7	-	-	-	-	245	54.3	63.8	73.6
**Time baseline until ESRD [years]**	403	-	-	-	-	158	7.2	12.0	17.0	-	-	-	-	245	6.1	11.7	16.7
**BMI [kg/m** ^**2**^ **]**	185,290	99,697	20.9	23.3	26.6	158	23.6	27.0	30.1	85,190	22.9	24.9	27.2	245	24.2	265	28.7
**Blood glucose [mmol/L]**	184,041	99,036	4.4	5.0	6.3	153	4.7	6.4	9.7	84,619	4.5	5.1	6.2	233	4.8	6.3	8.4
**Systolic blood pressure [mmHg]**	185,285	99,723	110.0	121.0	140.0	158	130.0	150.0	165.0	85,215	120.0	130.0	140.0	244	130.0	150.0	160.0
**Diastolic blood pressure [mmHg]**	185,189	99,635	70.0	80.0	85.0	158	80.0	90.0	100.0	85,152	75.0	80.0	90.0	244	80.0	90.0	100.0
**Total cholesterol [mmol/L]**	182,932	98,114	4.7	5.4	6.2	155	5.6	6.3	7.6	84,421	4.7	5.5	6.4	242	5.4	6.2	7.2
**Triglycrides [mmol/L]**	182,729	98,002	0.8	1.1	1.5	155	1.2	1.6	2.3	84,330	1.0	1.4	2.1	242	1.4	2.0	3.2
**Gamma-GT [U/L]**	182,743	97,994	12.5	17.9	25.1	155	17.9	25.1	37.6	84,351	19.7	28.6	44.8	242	23.3	35.8	59.1

*Wilcoxon Signed-Rank Tests between the two groups ‚No ESRD‘ and ‚ESRD‘ revealed significant differences (*p<0*.*001*) for all parameters, both in women and in men.

During the whole study period 813 patients developed ESRD in Vorarlberg, of whom 403 (158 women and 245 men) had undergone a previous health examination. Mean age at the start of renal replacement therapy (RRT) was 63.4 (SD 12.7) years. Of ESRD patients 45.4% were overweight, 21.3% were obese and 35.7% were smokers. The ESRD incidence rates during the study period were 14.5 per 100,000 person-years for the whole population of Vorarlberg and 13.4 per 100,000 person-years for VHM&PP participants.

Hazard ratios (HR) of ESRD were calculated for all categorical variables in a basic model (adjusted for age, sex and smoking status) for the entire observation period. Apart from smoking and being underweight all other categorical variables were significantly associated with an increased risk of ESRD (Table 2). The fully adjusted model (adjusting the basic categorical model for all other available covariates) showed a significantly elevated risk for increasing age, male sex (*vs*. female, HR 1.51 (95% confidence interval (CI) 1.20–1.88)), smoking (*vs*. non-smoking, HR 1.30 (1.04–1.61)), prevalent diabetes (*vs*. normoglycaemia, HR 1.39 (1.10–1.76), prehypertension, hypertension stage I and stage II (*vs*. normal BP, HR 1.98 (1.08–3.64), HR 3.38 (1.84–6.23) and HR 7.50 (4.07–13.83), respectively) and higher triglyceride categories (Table 2).

**Table 2:** Associations between risk factors and end-stage renal disease.

			Basic model	Fully adjusted model
Risk Factor	Categories	n total	HR(95% CI)*	HR(95% CI)**
**Age**	< 30	52,669	1.00	1.00
**[years]**	30–39	44,427	3.72(2.17–6.39)	2.57 (1.48–4.44)
	40–49	34,820	8.47 (5.08–14.12)	4.08 (2.40–6.92)
	50–59	26,955	11.17 (6.69–18.67)	4.10 (2.38–7.05)
	60–69	16,682	20.31 (12.11–34.07)	6.48 (3.73–11.25)
	≥ 70	9,788	13.47 (7.08–25.62)	4.04 (2.05–7.95)
**Sex**	Women	99,881	1.00	1.00
	Men	85,460	1.91 (1.56–2.35)	1.51 (1.21–1.89)
**BMI**	< 18.5	5,956	0.26 (0.04–1.86)	0.40 (0.06–2.86)
**[kg/m** ^**2**^ **]**	≥ 18.5 and < 25	101,657	1.00	1.00
	≥ 25 and < 30	57,708	1.56 (1.24–1.96)	1.06 (0.83–1.36)
	≥ 30	19,969	2.34 (1.79–3.12)	1.20 (0.89–1.61)
**Smoking status**	Non-smoker	131,969	1.00	1.00
	Smoker	53,372	0.88 (0.62–1.27)	1.30 (1.04–1.63)
**Blood glucose**	< 5.551	118,715	1.00	1.00
**[mmol/L]**	≥ 5.551 and < 6.939	32,677	1.14 (0.86–1.51)	1.09 (0.82–1.44)
	≥ 6.939	32,649	1.68 (1.33–2.11)	1.37 (1.08–1.73)
**Hypertension**	Normal	117,658	1.00	1.00
	Prehypertension		2.12 (1.18–3.80)	1.98 (1.08–3.64)
	Stage 1 hypertension		4.26 (2.39–7.62)	3.38 (1.84–6.23)
	Stage 2 hypertension	67,506	10.46 (5.87–18.66)	7.50 (4.07–13.83-)
**Triglycerides**	< 1.17	88,513	1.00	1.00
**[mmol/L]**	≥ 1.17 and < 2.28	68,927	2.25 (1.71–2.96)	1.85 (1.39–2.47)
	≥ 2.28 and < 5.7	23,300	3.71 (2.75–5.00)	2.54 (1.83–3.54)
	≥ 5.7	1,989	8.56 (5.35–13.71)	5.27 (3.16–8.80)
**Total cholesterol**	< 5.18	74,106	1.00	1.00
**[mmol/L]**	≥ 5.18 and <6.216	59,518	1.31 (0.97–1.76)	1.08 (0.79–1.46)
	≥ 6.216	49,308	1.91 (1.44–2.54)	1.15 (0.85–1.55)
**Gamma-GT**	Normal	159,091	1.00	1.00
**[U/L]**	Men: ≥61 Women: ≥36	23,651	1.61 (1.28–2.02)	1.02 (0.80–1.30)

*Basic adjustment model, adjusted for age, sex and smoking status.

**Fully adjusted model including all metabolic factors simultaneously adjusted for age, sex and smoking status

As shown in Table 3, all basic models using continuous variables revealed a significantly increased risk for ESRD. In fully adjusted models, a significantly increased risk for the development of ESRD was found for all factors except GGT in the entire study population. Increasing TC concentrations increased ESRD risk (HR 1.22 (1.13–1.32) per 1 mmol/L increment).

**Table 3:** Associations between risk factors and end-stage renal disease for continuous data.

		Basic model	Fully adjusted model**		
Risk Factor		Total	Total 380/181,139	Women 150/97,311	Men 230/83,828
	Cases / n*	HR (95% CI)*			
**Smoking status**	403 / 185,341	1.42 (1.15–1.75)	1.33 (1.06–1.66)	1.26 (0.84–1.90)	1.32 (1.02–1.72)
**BMI [kg/m** ^**2**^ **]**	403/185,290	1.10 (1.08–1.12)	1.04 (1.01–1.06)	1.04 (1.01–1.07)	1.03 (0.99–1.07)
**Blood glucose [mmol/L]**	386/ 184,041	1.14 (1.10–1.18)	1.09 (1.05–1.12)	1.11 (1.05–1.17)	1.06 (1.02–1.11)
**Systolic blood pressure [5 mmHg]**	402/185,285	1.17 (1.15–1.19)	1.10 (1.07–1.14)	1.11 (1.06–1.16)	1.11 (1.07–1.15)
**Diastolic blood pressure [5 mmHg]**	402/185,189	1.30 (1.25–1.34)	1.09 (1.03–1.15)	1.12 (1.02–1.22)	1.07 (1.00–1.15)
**Triglycerides [mmol/L]**	397/182,729	1.21 (1.17–1.25)	1.07 (1.02–1.13)	1.03 (0.94–1.13)	1.11 (1.04–1.17)
**Total cholesterol [mmol/L]**	397/182,932	1.37 (1.29–1.45)	1.22 (1.13–1.32)	1.27 (1.13–1.43)	1.21 (1.10–1.34)
**Log_Gamma-GT [U/L]**	397/182,742	1.56 (1.37–1.78)	1.05 (0.90–1.22)	1.34 (1.06–1.69)	0.91 (0.74–1.11)

*Basic adjustment model, adjusted for age, sex and smoking status.

**Fully adjusted model including all metabolic factors simultaneously adjusted for age, sex and smoking status).

After stratification by sex, FBG, BPsys, BPdia and TC were predictive for both women and men. BMI and GGT were significantly associated with an increased ESRD risk for women only, smoking and TG for men only (Table 3).

Cox models for continuous exposures adjusted for all variables showed differences in HR for all risk factors dependent on 5-year time intervals between baseline and the diagnosis of ESRD (Table 4). A sex-specific evaluation revealed good consistency between men and women. Therefore, both were investigated together.

**Table 4:** Association between risk factors and end-stage renal disease for continuous data considering 5-year time intervals.

Follow-up time	≤ 5 years	5–10 years	10–15 years	≥ 15 years
Cases / n	67 / 181,13	84 / 174,730	93 / 142,011	136 / 109,255
	**HR (95% CI)** *			
**BMI [kg/m2]**	0.99 (0.93–1.05)	0.98 (0.93–1.04)	1.05 (1.00–1.10)	1.08 (1.04–1.12)
**Blood glucose [mmol/L]**	1.03 (0.94–1.13)	1.04 (0.96–1.12)	1.12 (1.05–1.20)	1.11 (1.05–1.17)
**Systolic blood pressure [5 mmHg]**	1.21 (1.15–1.29)	1.19 (1.13–1.26)	1.04 (0.98–1.11)	1.02 (0.97–1.08)
**Diastolic blood pressure [5 mmHg]**	0.99 (0.88–1.12)	0.96 (0.86–1.07)	1.28 (1.15–1.43)	1.13 (1.03–1.24)
**Triglycerides [mmol/L]**	1.07 (0.97–1.17)	1.09 (0.98–1.21)	1.06 (0.95–1.19)	1.08 (0.98–1.18)
**Total cholesterol [mmol/L]**	1.50 (1.31–1.71)	1.18 (1.00–1.40)	1.19 (1.02–1.40)	1.09 (0.94–1.25)
**Log_Gamma-GT [U/L]**	0.58 (0.38–0.87)	1.23 (0.91–1.67)	1.00 (0.73–1.37)	1.30 (1.01–1.67)

*Cox models including all metabolic factors simultaneously adjusted for, age, sex, smoking status) for each time interval.; HR: Hazard Ratios; CI: Confidence Interval.

BPsys and TC were associated with an increased risk for ESRD occurring up to 10 years after baseline. In contrast, factors predicting an increased risk for ESRD more than 10 years after baseline were smoking, BMI, BPdia, FBG and GGT.

## Discussion

In a large population-based cohort we identified age, male sex, smoking, elevated BMI, diabetes, arterial hypertension, elevated TG, TC and GGT levels as risk factors for ESRD development. The study provides evidence that specific potentially modifiable risk factors occur in time-dependent patterns long before the disease becomes clinically evident. The analysis identified high BPsys and TC as factors with a short-term effect of less than 10 years after baseline. In contrast, smoking as well as increases in BMI, FBG levels, BPdia and GGT were identified as long-term risk factors with a significant effect occurring after 10 years and longer after baseline. Additionally, a sex-specific risk pattern was observed with smoking and TG as significant predictors for men only, and BMI and GGT for women only.

The findings of Kastarinen et al. in a large Finnish prospective cohort study are consistent with our results [7]. They observed similar crude hazard ratios for the development of ESRD for diabetes, hypertension, obesity and male sex. However, no adjustments for these different covariates were made in their analysis. Hsu et al. focused on risk factors for ESRD during a 25-year follow-up in predominantly white and African American individuals [6]. Diabetes, hypertension and excessive weight were associated with an increased risk for ESRD in their adjusted model. We provide further evidence on the impact of these established cardiovascular risk factors in a large population-based European cohort.

Information about the time-dependent association between risk factor and the development of ESRD is sparse. A recently published study investigated mid-adulthood risk factors for the development of CKD in the Framingham Offspring cohort [9]. This case-control study calculated Odds Ratios for the effect of risk factors 10, 20 and 30 years before the diagnosis of CKD defined as eGFR ≤60 ml/min/1.73m^2^. Hypertension, obesity and elevated triglycerides 30 years prior to the diagnosis of CKD showed significantly increased OR, when adjusting for age, sex and time period. Our study extends these findings of a time-dependent risk pattern in relation to long-term risk factors that are present many years before the clinical transitional event from the intermediate of a moderately decreased kidney function to dialysis-dependent ESRD. The duration of risk factor exposure may play an important role in the progression of ESRD later in life.

Obesity is a well-characterised risk factor for CKD and ESRD. Recently, we were able to assess elevated BMI early in life as a predictor of lower glomerular filtration rate (GFR) and higher urinary albumin creatinine ratio (ACR) in men up to more than 20 years later [10]. A large retrospective population-based cohort study from Israel evaluated the association between BMI at age 17 and later incident ESRD during a 25-year follow-up [22]. Our observation that elevated BMI as well as obesity were significantly associated with increased ESRD risk underlines the importance of BMI in adolescence and mid-adulthood as a risk factor for renal failure in later life. Our observation that an increased risk associated with BMI becomes evident after 10 years indicates that not only the extent but also the duration of obesity may play an important role. A recent study described an association between ESRD and higher BMI only in those with the metabolic syndrome whereas obesity without the metabolic syndrome was actually protective [23]. Unfortunately, we cannot distinguish between subjects with or without the metabolic syndrome in our database and therefore we are unable to prove whether this is also the case in our cohort. This study, however, had a mean observation period of six years and included many black individuals. We found that the association between high BMI and ESRD only became positively significant after more than ten years. We speculate that the effect of obesity, even in the absence of the metabolic syndrome, on kidney function is really long-term or that many obese individuals may have developed the metabolic syndrome over the years.

Hypertension is an established and strong independent risk factor for ESRD in men and women [8]. Even modest elevations in blood pressure are associated with an increased incidence of ESRD [24]. Our study confirms these results. We found that prehypertension doubles the risk for ESRD compared to normal blood pressure. A large prospective cohort study including 332,544 male participants identified a greater estimated risk for ESRD for increased BPsys as compared with elevated BPdia [25]. However, this analysis was performed in male participants only and a modification effect by follow-up time was not considered. Our findings suggest that the impact of BPsys and BPdia on renal function may indeed be time-dependent. While elevated diastolic pressure was a better predictor of ESRD more than ten years after baseline, BPsys became more associated with ESRD less than ten years after baseline. One explanation for this observation could be that BPdia decreases with age and progressive arterial stiffening, which is especially prominent in CKD, and thus loses its predictive value for ESRD with increasing age and progressing CKD.

Hsu et al. and Kastarinen et al. did not observe an association between TC levels and risk for ESRD over a 25-year period [6, 7]. In our study, higher TC levels were predictive of future ESRD in the full adjustment model with continuous variables. Our time window analysis, however, showed that the effect of hypercholesterolemia was highest in the first five years after the HE. Therefore, our results do not contradict other studies. The lack of a long-term association between hypercholesterolemia and ESRD may possibly be caused by a high risk for cardiovascular death in patients with high TC. These patients may not live long enough to develop ESRD [26]. Alternatively, hypercholesterolemia in the years before ESRD may be caused by progressive CKD and nephrotic proteinuria, for example in diabetic nephropathy, and may therefore be considered a consequence rather than a cause of progressive kidney disease. This view is supported by a recent report from the Chronic Renal Insufficiency Cohort (CRIC) study showing that TC levels are not associated with CKD progression over a period of four years [27].

Data on triglyceride levels and long-term risk for ESRD are scarce. The CRIC study found no association between triglycerides and progression of CKD [27]. In the Framingham Offspring study higher triglycerides were associated with future CKD [9]. Similarly, the Atherosclerosis Risk in Communities (ARIC) study found higher triglyceride levels to be predictive of a future decline in renal function [28]. Our results showing higher TG levels being associated with future ESRD, especially in men, would further support the findings of the two latter studies and confirm an association between hypertriglyceridemia and kidney disease.

Previous studies have identified the diagnosis of diabetes as a long-term predictor for CKD and ESRD [7, 9]. This is consistent with our study, which revealed an increased risk for ESRD with higher FBG, especially over the long-term after baseline, whereas the risk was not elevated when hyperglycaemia was diagnosed closer to ESRD. This observation would be in line with the role of hyperglycaemia in initiation but not progression of diabetic kidney disease.

As shown earlier, elevated GGT levels are considered a marker for non-alcoholic fatty liver disease and are associated with diabetes, arterial hypertension and cardiovascular diseases [29, 30], [31]. Recently, an association between elevated GGT and CKD has been described [10, 32, 33]. However, we are not aware of a study investigating the association between GGT and ESRD. We observed an increased GGT level to be a strong predictor for ESRD in women over the whole study period. This somehow contrasts our previous finding that elevated GGT was predictive of albuminuria and lower eGFR in men [10]. Clearly, whether GGT and non-alcoholic fatty liver disease are predictors of long-term risk for ESRD requires further research.

### Strengths and Limitations

The large study population of 185,341 participants with a wide age range and the long-term follow-up are key strengths of this study. The two data sets, namely VHM&PP and OEDTR, are qualitatively well described and attain large population coverage [15, 16]. Nevertheless, the fact that health-conscious people are more likely to attend voluntary health examinations may possibly introduce a selection bias. However, the comparable ESRD incidence rates between the total population and VHM&PP participants would argue against such a bias.

One limitation is the lack of information about medication and disease history, which could have resulted in uncontrolled confounding.

Another limitation is the lack of data about kidney function. Unfortunately, serum creatinine and proteinuria measurements were not part of the standard laboratory panel included in the regular health examinations. Reduced glomerular filtration rate and albuminuria are markers of established kidney disease. In a long-term population-based study as ours the primary goal is to identify not so much progression factors but also initiation factors for CKD and ESRD, and GFR and albuminuria levels are not so relevant.

The OEDTR includes only ESRD patients undergoing renal replacement therapy. Therefore, our study may miss patients with ESRD, who were not treated by dialysis or kidney transplantation.

In conclusion, anthropometric and metabolic factors are associated with the risk for ESRD in a sex- and time-dependent manner. This needs to be considered for future preventive and therapeutic strategies to reduce the incidence of ESRD. Whether early intervention to ameliorate these factors, in particular obesity, would reduce the incidence of CKD and ESRD is unproven, but seems very likely.
